# A Giant Left Atrial Myxoma Neovascularized from the Right Coronary Artery

**DOI:** 10.1155/2015/614830

**Published:** 2015-04-15

**Authors:** Demet Menekse Gerede, Irem Muge Akbulut, Sadık Ersoz, Mustafa Kilıckap

**Affiliations:** ^1^Department of Cardiology, Cebeci Heart Center, Ankara University School of Medicine, Cebeci, 06590 Ankara, Turkey; ^2^Department of Cardiovascular Surgery, Ankara University School of Medicine, Ankara, Turkey

## Abstract

Myxomas are benign and the most common tumors of the cardiac muscle (Reynen, 1995). They are predominantly located in the left atrium. Clinical manifestations may vary according to the localization and the size of the myxoma. On the other hand, imaging of a myxoma by contrast dye during coronary angiography is a rare sign, which displays the vascular supply of the tumor. Here, we report the case of a 51-year-old man presenting with presyncope and palpitations due to a giant left atrial myxoma having its vascular supply from the right coronary artery (RCA).

## 1. Case Presentation

**Figure 1:**
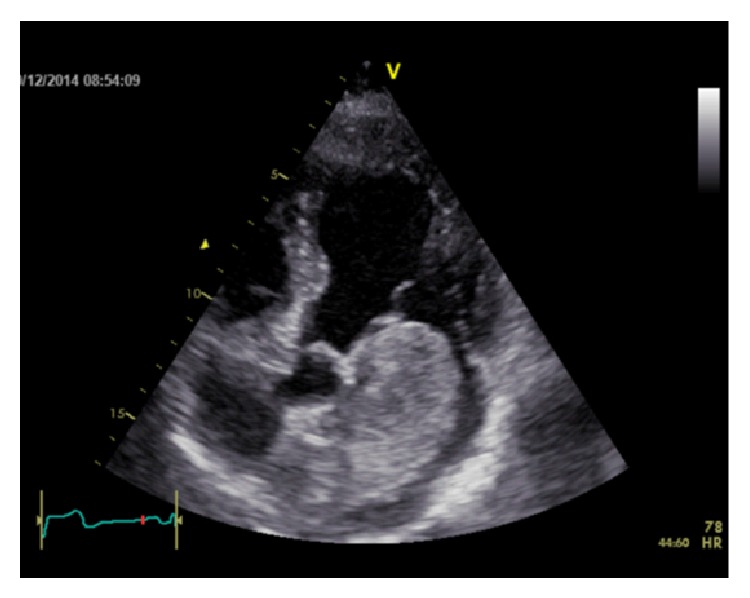
Transthoracic echocardiography shows the giant left atrial myxoma protruding through the mitral valve into the left ventricle during diastole.

**Figure 2:**
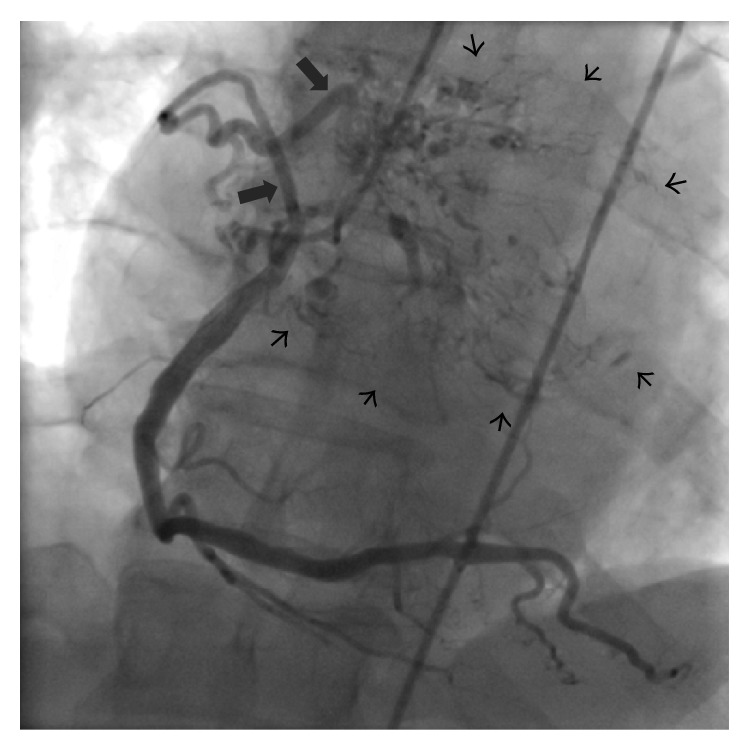
Coronary angiography image with a conus branch of the right coronary artery (large black arrows) vascularizing the myxoma with a wide area vascular network (small black arrows).

**Figure 3:**
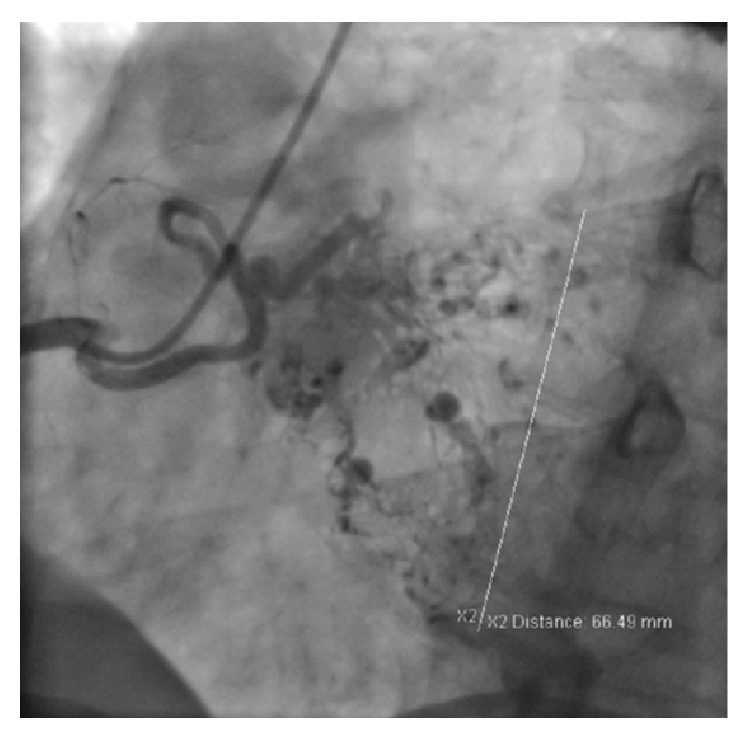
Right coronary angiogram with zoom mode shows diameter of left atrial myxoma vascularity by conus branch.

A 51-year-old man was referred to cardiology clinic due to presyncope attacks and palpitations. His medical history included type 2 diabetes mellitus, hypogonadism of unknown etiology, and hypertension. His physical examination was unremarkable. A transthoracic echocardiography was performed and revealed a giant left atrial mass attached to the interatrial septum with dimensions of 4.7 × 6.7 cm (Figure 1 and Video 1 in Supplementary Material available online at http://dx.doi.org/10.1155/2015/614830), resulting in an average mitral transvalvular gradient of 8 mmHg. Echocardiographic findings indicated a suspicious diagnosis of left atrial myxoma. A preoperative coronary angiography was performed and revealed normal coronary arteries and the neovascularization of the mass from the conus branch of the right coronary artery (Figures 2 and 3). No other imaging study was performed prior to the surgery. The patient underwent surgery, which was carried out under cardiopulmonary bypass. A portion of the mass was excised by left atriotomy. The rest of the mass was removed by an incision to the interatrial septum. No attempts to mitral valve were performed during surgery.

The histopathologic examination of the mass was compatible with myxoma. A transesophageal echocardiography was performed after the surgery and showed an intact interatrial septum and mild mitral regurgitation. One week after the surgery, the patient was in good health and discharged from the hospital.

## 2. Discussion

Myxomas are the most frequent benign tumors of the heart. Approximately 85% of the myxomas are located in the left atrium [1]. The clinical manifestations usually depend on the anatomic position and size of the mass. There are mainly 3 types of presentations: embolic, obstructive, and constitutional. Embolic manifestations include visceral infarctions, stroke, and myocardial infarction. Obstructive manifestations are usually mistaken for valvular stenosis. For instance, mitral valve obstruction due to left atrial myxoma presents with symptoms such as syncope and dyspnea, mimicking mitral valve stenosis. It was previously shown that mitral stenotic effects occurred when the diameter of the mass exceeded 5 centimeters [2]. In our case the diameter of the tumor was 6.7 cm, causing moderate mitral transvalvular gradient and presyncope attacks. Finally, myxomas may be associated with constitutional symptoms such as weight loss, malaise, fever, and loss of appetite.

Beyond its value in ruling out coronary artery disease before surgery, a coronary angiography is also crucial for detecting the neovessels of the tumour. In the majority of cases, the supply of the vascularization is the circumflex artery, followed by the right coronary artery [3]. Similarly in our case, the right coronary artery was the source of the neovascularization.

Only a few patients with giant left atrial myxoma supplied from RCA were demonstrated [4–6]. Our patient, presenting with obscure symptoms, is a rare case of a giant left atrial myxoma, which was shown with contrast dye on RCA angiogram.

## 3. Conclusion

In conclusion, myxomas are rare, benign cardiac tumours. They can be presented with symptoms of obstruction when they are large in size, as in our case. Neovascularization can be seen in myxomas so that preoperative coronary angiograms should be performed in order to check for the coronary supply of the mass.

## Supplementary Material

Video 1: Three-dimensional transthorasic echocardiography showing mobile left atrial myxoma.
